# Supplementary material for: Differences in the Shiga Toxin (Stx) 2a Phage Regulatory Switch Region Influence Stx2 Localization and Virulence of Stx-Producing Escherichia coli in Mice
Source: Microorganisms. 2023 Jul 28;11(8):1925. doi: 10.3390/microorganisms11081925 (PMC10458857; doi:10.3390/microorganisms11081925)
Supplement: Supplementary file 1 [file microorganisms-11-01925-s001.zip › Supplemental figure S1.pdf]

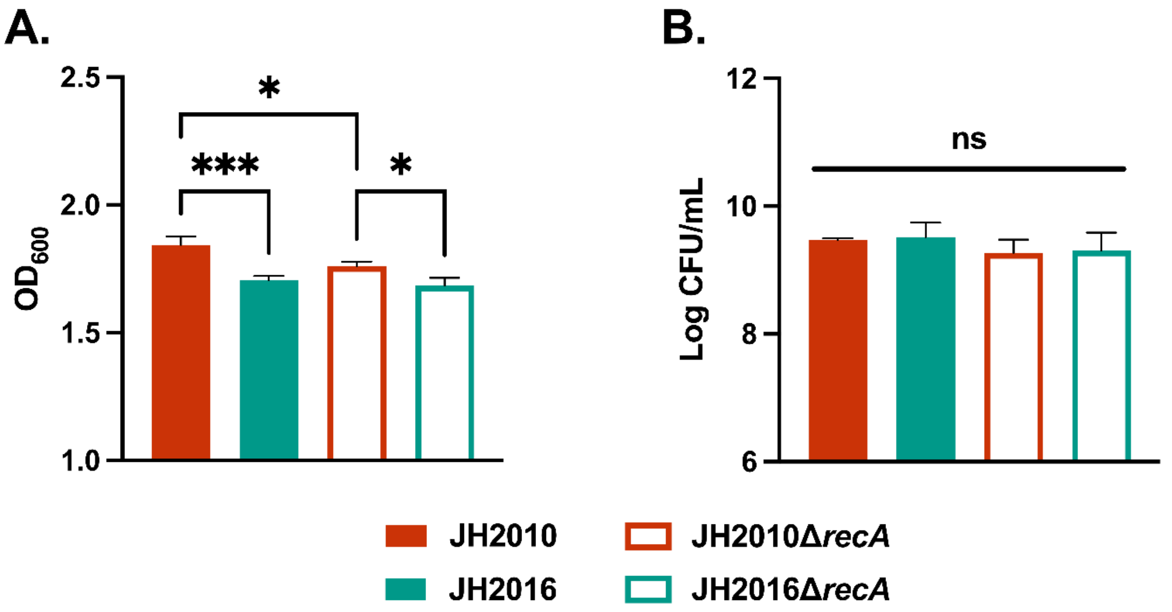

Figure S1. ***In vitro* growth of wild-type and *recA* mutant strains.** Overnight cultures grown in LB-YE of JH2010, JH2016, and the corresponding *recA* mutant strains, were assayed for growth by measuring (A) the optical density at 600 nm (OD_{600}) or by (B) measuring the number of colony forming units (CFU) per mL of culture. Significance was calculated by one-way ANOVA with Šídák's test for multiple comparisons. *$P < 0.05$, ***$P < 0.001$.
