# Supplementary material for: Differences in the Shiga Toxin (Stx) 2a Phage Regulatory Switch Region Influence Stx2 Localization and Virulence of Stx-Producing Escherichia coli in Mice
Source: Microorganisms. 2023 Jul 28;11(8):1925. doi: 10.3390/microorganisms11081925 (PMC10458857; doi:10.3390/microorganisms11081925)
Supplement: Supplementary file 1 [file microorganisms-11-01925-s001.zip › Supplemental figure S2.pdf]

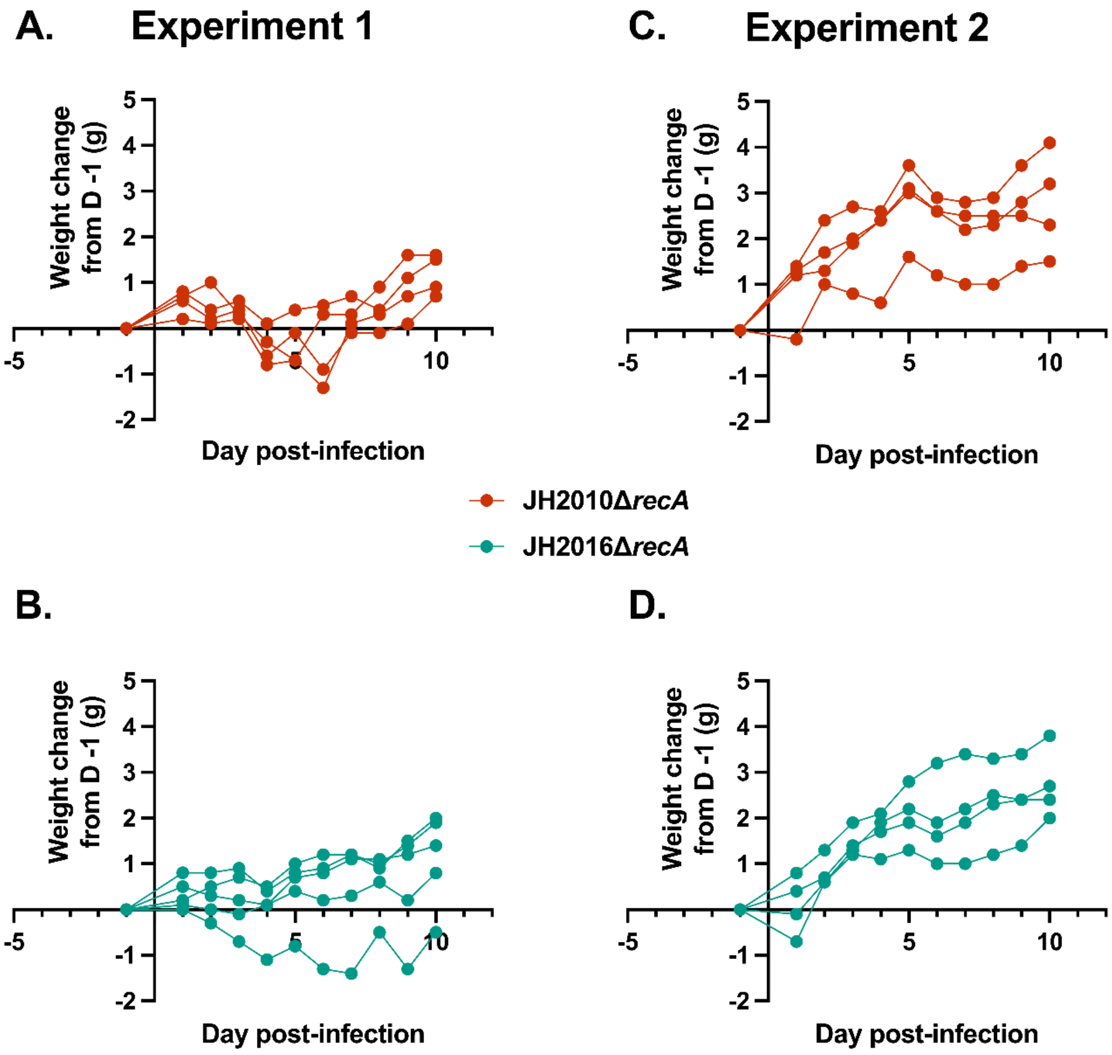

Figure S2. ***In vivo recA*** **mutant strain mouse weights.** Str-treated mice were infected with either JH2010Δ*recA* or JH2016Δ*recA* in individual experiments 1 (A,B) and 2 (C,D). Daily weights for individual infected mice were measured starting at day 1 (D1) post-infection, and are displayed as the difference in weight from 24 hours pre-infection (D-1).
