# Supplementary material for: Differences in the Shiga Toxin (Stx) 2a Phage Regulatory Switch Region Influence Stx2 Localization and Virulence of Stx-Producing Escherichia coli in Mice
Source: Microorganisms. 2023 Jul 28;11(8):1925. doi: 10.3390/microorganisms11081925 (PMC10458857; doi:10.3390/microorganisms11081925)
Supplement: Supplementary file 1 [file microorganisms-11-01925-s001.zip › Supplemental figure S3.pdf]

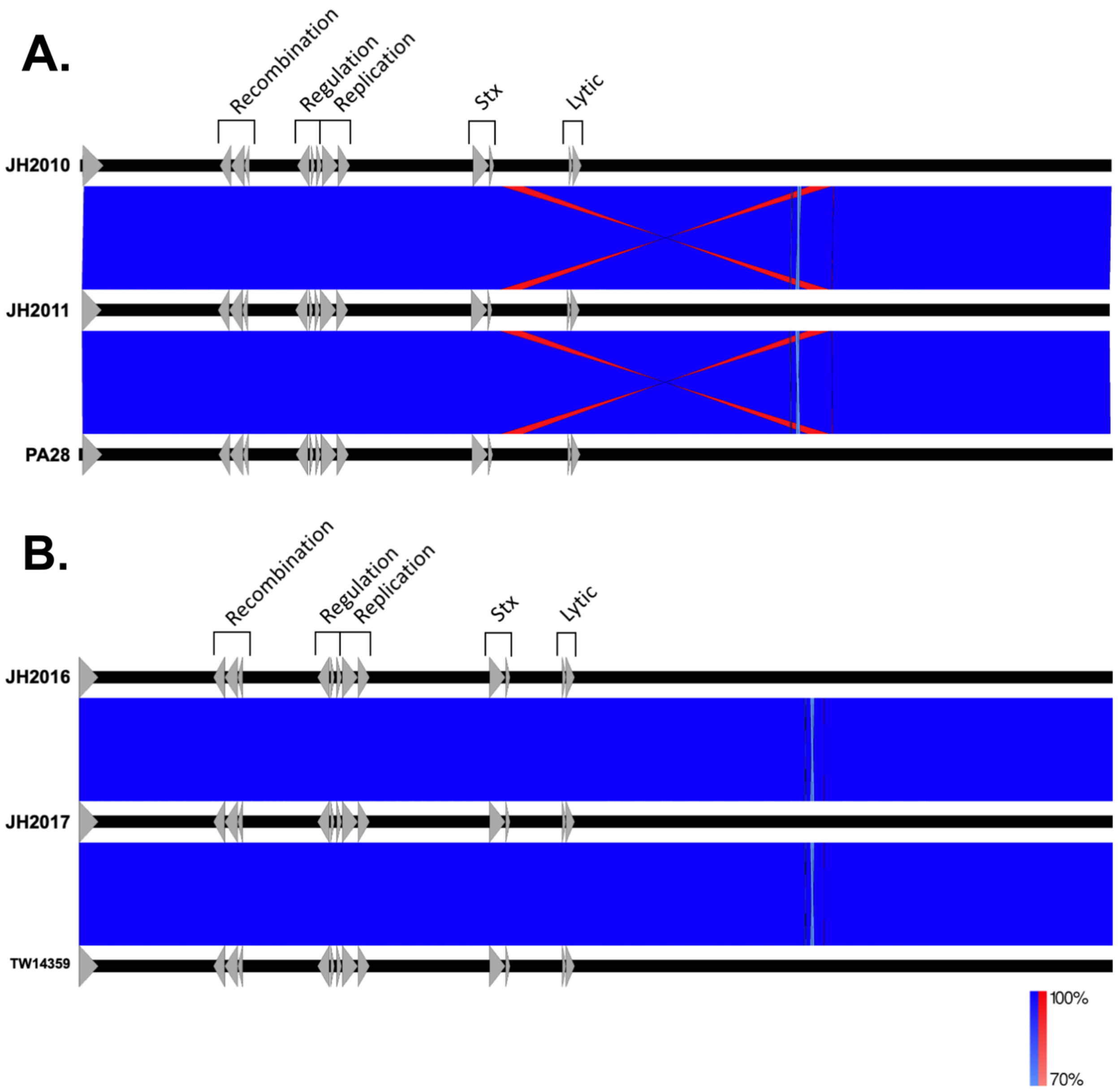

Figure S3. Pairwise alignment of JH2010-like and JH2016-like *stx*$_{2a}$-phages. NCBI Blast alignment of the *stx*$_{2a}$-phages from (A) JH2010-like strains and (B) JH2016-like strains were visualized using Easyfig. Gray arrows represent relevant functional clusters of genes based on gene product annotation within the labelled phage regions. The saturation of the color indicates percent homology between the two sequences; blue blocks represent sequences in the same orientation, while red blocks represent sequences in the opposite orientation. White regions represent areas with less than 70% identity.
