# Supplementary material for: Differences in the Shiga Toxin (Stx) 2a Phage Regulatory Switch Region Influence Stx2 Localization and Virulence of Stx-Producing Escherichia coli in Mice
Source: Microorganisms. 2023 Jul 28;11(8):1925. doi: 10.3390/microorganisms11081925 (PMC10458857; doi:10.3390/microorganisms11081925)
Supplement: Supplementary file 1 [file microorganisms-11-01925-s001.zip › Supplemental figure S4.pdf]

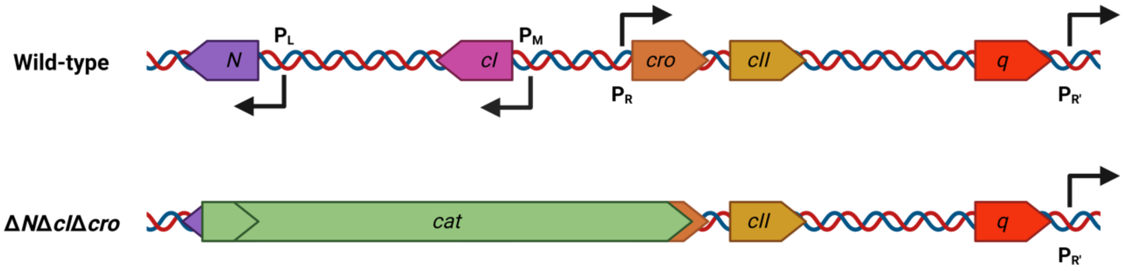

Figure S4. Diagrams of the *N* to *q* region of the wild-type *stx*-phage and the deletion mutant strains. The wild-type diagram (not to scale) shows the relative locations of genes encoding key regulatory proteins (*N*, *cI*, *cII*, *cro*, *q*) and elements (promoters P_L, P_M, P_R, $P_{R'}$) on the *stx*-phage. The Δ*N*Δ*cI*Δ*cro* diagram shows the predicted deletion of the specified regulatory genes through lambda Red-mediated recombination by insertion of a chloramphenicol resistance cassette (*cat*).
