# Supplementary material for: Differences in the Shiga Toxin (Stx) 2a Phage Regulatory Switch Region Influence Stx2 Localization and Virulence of Stx-Producing Escherichia coli in Mice
Source: Microorganisms. 2023 Jul 28;11(8):1925. doi: 10.3390/microorganisms11081925 (PMC10458857; doi:10.3390/microorganisms11081925)
Supplement: Supplementary file 1 [file microorganisms-11-01925-s001.zip › Supplemental figure S5.pdf]

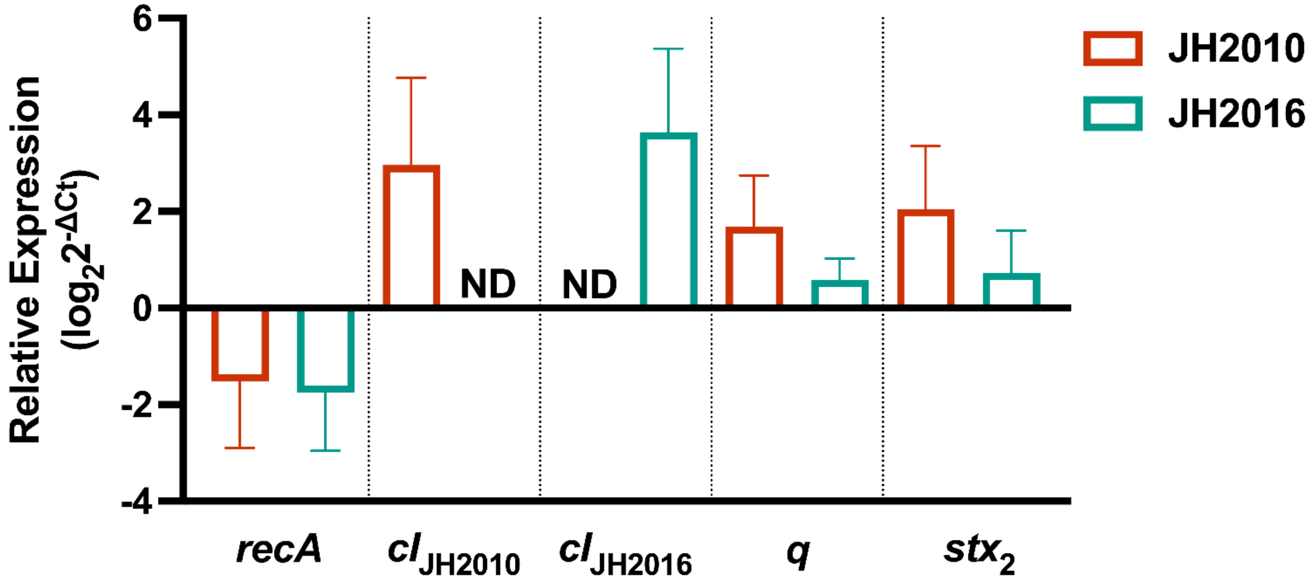


**Figure S5. Gene expression of wild-type JH2010 and JH2016 by RT-qPCR.** Relative transcription of genes from overnight LB-YE cultures of JH2010 and JH2016 as measured by RT-qPCR. Results are graphed as mean log fold-change from *gyrB* transcript ± SD (*n* = 8). Significance was calculated by one-way ANOVA with Šídák's test for multiple comparisons.
