# Supplementary material for: Differences in the Shiga Toxin (Stx) 2a Phage Regulatory Switch Region Influence Stx2 Localization and Virulence of Stx-Producing Escherichia coli in Mice
Source: Microorganisms. 2023 Jul 28;11(8):1925. doi: 10.3390/microorganisms11081925 (PMC10458857; doi:10.3390/microorganisms11081925)
Supplement: Supplementary file 1 [file microorganisms-11-01925-s001.zip › Supplemental table S1.pdf]

Table S1: Identity between *stx*$_{2a}$-phage regulatory gene and encoded protein sequences from JH2010 and JH2016. Regulatory genes and predicted proteins were identified based on location within the phage genome and synteny to lambda phage, as well as NCBI annotation. Percent nucleotide and amino acid identity were calculated using Clustal Omega.

**Regulatory Gene/Protein**	**% Nucleotide Identity**	**% Amino Acid Identity**
***N*/N**	38.3	15.3
***cI*/CI**	41.1	16.9
***cro*/Cro**	39.7	23.4
***cII*/CII**	88.6	86.7
