# Supplementary material for: Differences in the Shiga Toxin (Stx) 2a Phage Regulatory Switch Region Influence Stx2 Localization and Virulence of Stx-Producing Escherichia coli in Mice
Source: Microorganisms. 2023 Jul 28;11(8):1925. doi: 10.3390/microorganisms11081925 (PMC10458857; doi:10.3390/microorganisms11081925)
Supplement: Supplementary file 1 [file microorganisms-11-01925-s001.zip › Supplemental table S2.pdf]

TABLE S2: Appearance of cI_{JH2010} and cI_{JH2016} sequences within the PulseNet reference library. NCBI Blast queries were conducted using cI_{JH2010} and cI_{JH2016} nucleotide sequences to compare within the PulseNet NCBI database (BioProject #PRJNA218110). The presence of *stx* subtypes and location of the *cI* genes was determined by NCBI annotation.

CI type	Strain Name (Accession #)	Accession #	Type(s) of *stx* genes present	Location of *cI*
cI_{JH2010}	*Escherichia coli* strain 08-3914 (parental of JH2011)	CP034808.1	stx_{2a}, stx_{2c}	stx_{2a}-phage
	Escherichia coli strain 2014C-3338	CP027452.1	stx_{1a}, stx_{2a}	stx_{1a}-phage
	Escherichia coli strain 2013C-4404*	CP027376.1	stx_{1a}	stx_{1a}-phage
	Escherichia coli strain 2013C-4390*	CP027484.1	stx_{1c}	stx_{1c}-phage
	Escherichia coli strain 2014C-3741	CP027328.1	stx_{1c}, stx_{2b}	stx_{1c}-phage
cI_{JH2016}	*Escherichia coli* strain PNUSAE013304*	CP034936.1	stx_{2a}, stx_{2c}	stx_{2a}-phage
	Escherichia coli strain 2009C-4687	CP034799.1	stx_{2a}, stx_{2c}	stx_{2a}-phage
	Escherichia coli strain 2010C-3142 (parental of JH2017)	CP034801.1	stx_{2a}, stx_{2c}	stx_{2a}-phage
	Escherichia coli strain 2017C-4109	CP030767.1	stx_{2a}, stx_{2c}	stx_{2a}-phage
	Escherichia coli strain 97-3250	CP027599.1	stx_{1a}, stx_{2a}	stx_{1a}-phage
	Escherichia coli strain 2014C-3011	CP027591.1	stx_{2c}	Non-*stx*-phage
	Escherichia coli strain 2013C-3264	CP027544.1	stx_{1a}	Non-*stx*-phage
	Escherichia coli strain 2013C-3252	CP027442.1	stx_{1a}	stx_{1a}-phage

	Escherichia coli strain 2011C-4251	CP027388.1	stx_{1a}, stx_{2a}	stx_{1a}-phage
	Escherichia coli strain 2015C-3101	CP027221.1	stx_{1a}, stx_{2a}	stx_{2a}-phage
	Escherichia coli strain 2015C-3163	CP027219.1	stx_{1a}	stx_{1a}-phage
	Escherichia coli strain 2011C-4315	CP024479.1	stx_{1a}	stx_{1a}-phage
	Escherichia coli strain 2013C-4465	CP015241.1	stx_{1a}	stx_{1a}-phage
	Escherichia coli strain 90-3040	CP027459.1	stx_{2a}	stx_{2a}-phage
	Escherichia coli strain 2013C-4538	CP027582.1	stx_{1a}	stx_{1a}-phage
	Escherichia coli strain 2014C-3050	CP027472.1	stx_{1a}	stx_{1a}-phage
	Escherichia coli strain 2014C-3599	CP027435.1	stx_{2a}	stx_{2a}-phage
	Escherichia coli strain 2014C-3655	CP027351.1	stx_{2a}	stx_{2a}-phage
	Escherichia coli strain 2015C-3107	CP027317.1	stx_{2a}	stx_{2a}-phage
	Escherichia coli strain 2014C-4423	CP027454.1	stx_{2a}	stx_{2a}-phage
	Escherichia coli strain 2014C-3003*	CP027672.1	stx_{1c}	stx_{1c}-phage
	Escherichia coli strain 2013C-3033	CP027323.1	stx_{1a}, stx_{1c}	stx_{1a}-phage
	Escherichia coli strain 2017C-4173W12	CP030768.1	None	Non-*stx*-phage

* Strains that did not have NCBI annotation data. For these strains, VirulenceFinder (at 95% identity, 80% coverage) was used to determine the types of *stx* genes present, and proximity of the *cI* nucleotide query to the identified *stx* genes was used to determined the location
